# RETIREMENT ADJUSTMENT: COMPARISON AMONG CHINESE, JAPANESE, AND KOREAN RETIREES

**DOI:** 10.1093/geroni/igz038.471

**Published:** 2019-11-08

**Authors:** Dannii Yeung, Takeshi Nakagawa, Jinmyoung Cho

**Affiliations:** 1 City University of Hong Kong, Hong Kong, Hong Kong; 2 National Center for Geriatrics and Gerontology, Aichi, Japan; 3 Baylor Scott & White Health, Temple, Texas, United States

## Abstract

Moving into retirement is a stressful life event. However, recent research findings suggest that not every retiree experiences maladjustment. This paper investigated longitudinal changes in depressive symptoms during the retirement transition utilizing harmonized data of CHARLS, JSTAR, and KLoSA. Participants were selected for examination if meeting the following criteria: They were working in Wave 1 and had retired in Wave 2 (China=1053; Japan=184; Korea=706). These participants were categorized into two subgroups based on their status in Wave 3, either remained retired or reemployed. The proportion of retirees who were reemployed in Wave 3 is significantly higher in Korea (49.3%) and China (41.1%) than in Japan (17.4%). In each wave, the level of depressive symptoms was measured by the 10-item CES-D scale. Results of the repeated measures analyses show that, even after controlling for gender, mean level of depression increased over time among Korean retirees [F(2,702)=3.65,p=.026,η2=.010], whereas Japanese retirees’ depressive symptoms only increased in Wave 2 (Mean Difference =.83,SE=.39,p=.034) but not in Wave 3. Among Chinese, depressive symptoms did not significantly worsen after retirement, but the changes varied between retired persons and those who were reemployed in Wave 3 [F(1,1030)=4.25, p=.040, η2=.004]. Specifically, individuals who were reemployed after retirement experienced a reduction in depressive symptoms between Wave 1 and 3, whereas a reverse pattern is shown among retirees. This suggests that reemployment is beneficial to the well-being of Chinese retirees. The effects of socioeconomic factors (e.g., private and public health insurance and pension support) on depressive symptoms will be discussed.

